# Effect of uterine peristalsis at the time of embryo transfer on the outcomes of IVF cycles: a prospective cohort study

**DOI:** 10.1186/s12958-026-01587-0

**Published:** 2026-07-09

**Authors:** Karim S Abdallah, Kamal M Zahran, Mahmoud A Othman, Ahmed Nasr

**Affiliations:** Department of Obstetrics and Gynecology, Faculty of Medicine, Women’s Health Hospital, Assiut University, Assiut, Egypt

**Keywords:** Uterine peristalsis, IVF, ICSI, Embryo transfer

## Abstract

**Background:**

It has recently been discovered that one of the key elements influencing endometrial receptivity is uterine peristalsis. This was a prospective cohort study aiming to assess uterine peristalsis at the time of embryo transfer in women undergoing IVF.

**Methods:**

Uterine peristaltic waves were assessed using transvaginal ultrasound by recording a 3-minute video to determine the frequency and the direction of peristalsis. Primary outcome was clinical pregnancy while secondary outcomes were biochemical pregnancy, miscarriage, ectopic pregnancy and live birth. Outcomes were compared in women who had no peristalsis (Absent group), had one or two waves per minute (< 3 Group), or those who had 3 or more waves per minute (≥ 3 Group).

**Results:**

Between August 2023 and January 2025, 127 women were included in this study, of whom 76 (59.8%) were fresh cycles and 51 (40.2%) were frozen cycles. The median number of peristaltic waves was one per minute. Peristalsis was absent in 34 women, < 3 in 72 women and ≥ 3 in 21 women. There was no statistically significant difference in pregnancy rate between the three groups (26.1% in absent group, 36.1% in < 3 group, and 33.3% in ≥ 3 group). The direction was cervico-fundal in 62 women, fundo-cervical in 5 women, and random in 26 women, with pregnancy rates 22%, 0% and 11%, respectively, with no statistically significant difference.

**Conclusion:**

We did not find a significant effect of the number or the direction of uterine peristalsis on any of the clinical outcomes of IVF. Therefore, we do not recommend its routine assessment before embryo transfer until further evidence is available.

**Trial registration:**

The study protocol was registered on 14 September 2023 at ClinicalTrials.gov (Registration number NCT06036459).

**Supplementary Information:**

The online version contains supplementary material available at 10.1186/s12958-026-01587-0.

## Background

Over time, there has been improvement in the utilization of assisted reproductive technologies (ART). Successful implantation is still difficult, though. It has recently been discovered that one of the key elements influencing endometrial receptivity is uterine peristalsis, or the rhythmic peristalsis of the sub-endometrial layer of the myometrium [1]. Clinical pregnancy rates are substantially lower in women with higher peristalsis rates (more than two waves per minute) [2].

Furthermore, women with lower peristaltic frequencies of 2 waves per minute had significantly higher odds of clinical pregnancy (OR 10.8) than women with higher frequencies of 4 waves per minute, according to a study examining peristaltic activity in frozen embryo transfer cycles [3].

A study by Vidal and colleagues showed that endometriosis had altered endometrial peristalsis using Cine magnetic resonance imaging (Cine-MRI) [4]. This mechanism could explain the low fecundity and lower pregnancy rates in women with endometriosis, demonstrating the broader clinical importance of the disorder. Adenomyosis and other conditions also show changed uterine peristalsis [5].

Additionally, peristaltic movements within the lesions (endometrial configuration changes, irregularities in signal conduction) during the luteal phase and hyperperistalsis in certain patients were reported in the study of patients with adenomyosis on cine-MRI [6].

The use of an oxytocin receptor antagonist during FET cycles in the adenomyosis population was linked to a lower rate of early miscarriage, which is believed to be caused by the attenuation of uterine dysfunctional peristalsis, according to a recent propensity scores matched study [7]. Atosiban, an oxytocin antagonist, was administered to the target population during FET cycles in a randomized control trial from Thailand [8]. Uterine peristalsis frequency was assessed before and after treatment, but the trial found no discernible differences in the percentage of patients who achieved implantation and clinical pregnancy between the atosiban and placebo groups.

This highlights how important it is to gain a better understanding of both the impacts of uterine contractility and the implantation process. Therefore, our study’s goal is to investigate the connection between frequency and direction of uterine peristaltic activity and clinical outcomes of IVF.

## Methods

### Study design

The study was a prospective cohort study that enrolled women who intended to have IVF/ ICSI cycles at Assisted Reproduction Unit of Women’s Health Hospital, Assiut University, Egypt.

The study protocol was registered at ClinicalTrials.gov (Registration number NCT06036459). Institutional Review Board of Faculty of Medicine, Assiut University approved the study protocol before starting enrollment (IRB approval number: 04-2023-100097).

All couples had proper counselling and signed an informed consent for participating in the study.

### Participants

Women were assessed for eligibility after oocyte retrieval and before embryo transfer during an IVF cycle. Women aged between 20 and 40 years old who had at least 1 good quality embryo for transfer, whether fresh or frozen cycles, were included in the study. Women with any uncorrected uterine pathology, such as uterine septum, polyp or adenomyosis, hydrosalpinx except if surgically removed or disconnected from the uterus, and thin endometrium (< 7 mm) at the time of embryo transfer were excluded from the study.

### IVF procedure

All steps for IVF/ICSI procedure either fresh or frozen thawed cycles, from the beginning of the cycle until just before the procedure of ET, have done as routinely decided according to the local protocol of the ART Unit at Women’s Health Hospital, Assiut University. In the day of ET, number and quality of Embryos have been decided according to the routine practice guided by the local protocol.

### Assessment of uterine peristalsis

Transvaginal ultrasonography scans were performed using the ultrasound machine Samsung SonoAce R5. In lithotomy position, assessment of uterine peristalsis was performed immediately before embryo transfer. The vaginal probe was introduced gently to avoid stimulating the uterus. The probe was fixed in the mid sagittal plane of the uterus, then a three-minute video was recorded.

Records were analyzed using a video player (VLC player). Videos were played at a speed 4 times faster than the regular speed. At this speed, uterine peristalsis was clearer and easier to be calculated. Two observers (MO and KA) assessed the records independently. Whenever there were conflicting results, the video was played by the two operators simultaneously until reaching consensus.

The uterine peristaltic waves were recorded as number of occurrences, in addition to their directions. The directions were either cervico-fundal (CF), fundo-cervial (FC), random (when the direction occurred at different sites of the myometrium at the same time, or when the direction differed among the peristaltic waves in the same woman), or absent.

### Outcomes

The primary outcomes were relation between the number and direction of uterine peristalsis and clinical pregnancy, defined as the presence of intrauterine fetal cardiac pulsations on ultrasound examination after 2 weeks or more from a positive pregnancy test.

Secondary outcomes were the relation between the number and direction of uterine peristalsis and the following: biochemical pregnancy (defined as a positive serum pregnancy test), miscarriage (defined as pregnancy loss after a positive pregnancy test and until 28 weeks of gestation), and live birth (defined as the delivery of one or more living babies at or after 28 weeks of gestation).

### Statistical analysis

Zhu 2014 reported a drop in clinical pregnancy rate in women with marked peristalsis (≥ 3 / minute) to 6% from 50% in women with less than 3 peristaltic waves per minute [9]. We have chosen a more conservative estimate of drop-in pregnancy rate in women with marked peristalsis from 50% to 25%. Using two-sided significance level of 90% at 80% power, Sample size was calculated to be 106 to detect a drop of pregnancy rate from 50% to 25%. Accounting for 20% drop-out rate, we planned to recruit 127 women.

The statistical analysis was done using Posit team (2025), RStudio: Integrated Development Environment for R. Posit Software, PBC, Boston, MA. Qualitative data were described as frequency and percentage. Quantitative data were described as mean ± standard deviation, or median with inter-quartile range. Chi square test was used for categorical variables, to compare between different groups. The Shapiro-Wilk test was used to test the normality distribution of the continuous variables. The t-test was used to compare the differences among the normally distributed continuous variables, while the differences among continuous variables without normal distribution were compared using the Mann Whitney U-test. Significance of the obtained results was judged at the 5% level (P value < 0.5).

## Results

**Fig. 1:**
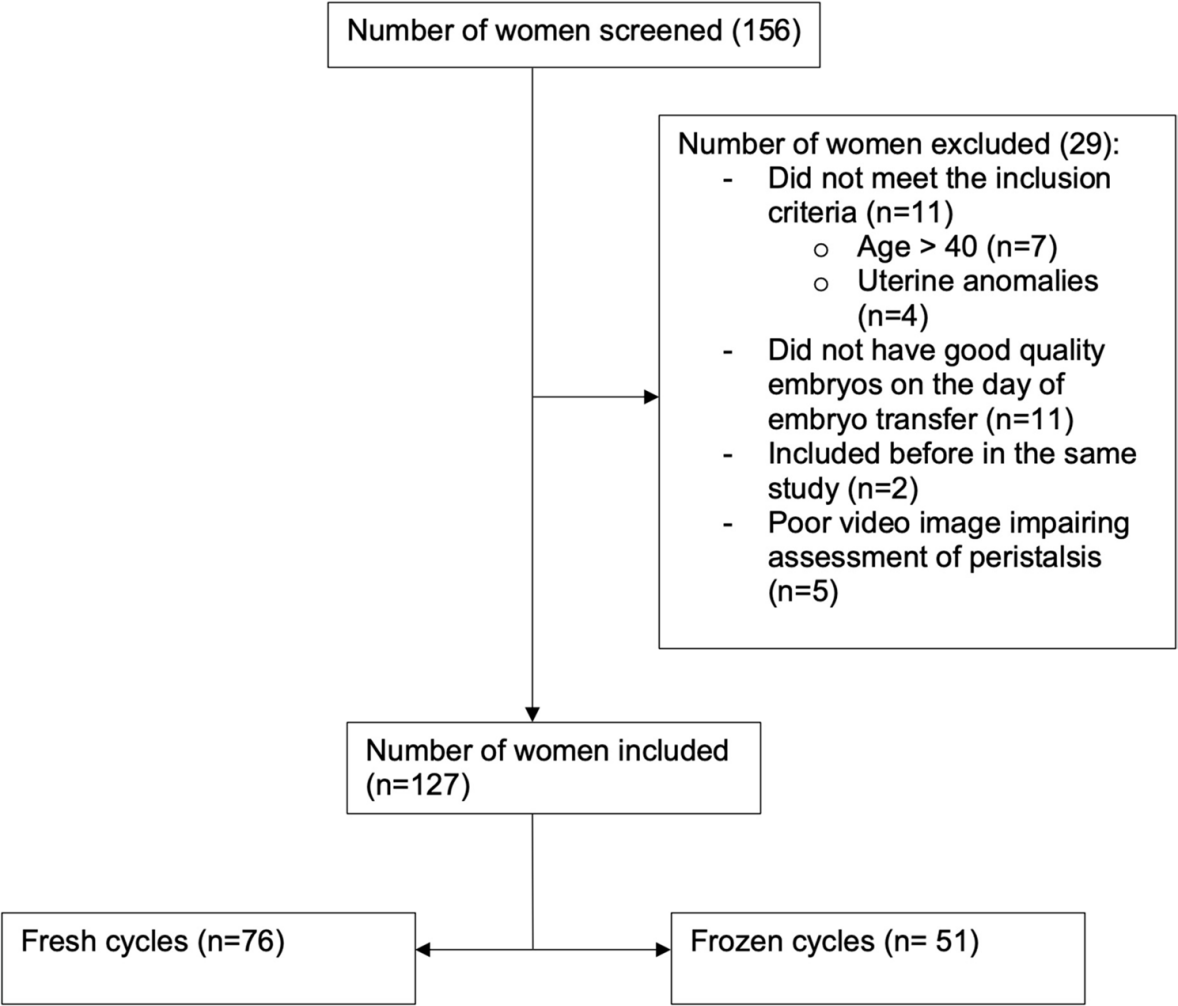
Study flow chart

From August 2023 until January 2025, 156 Women were assessed for eligibility, of which 127 women met the inclusion criteria. Twenty-nine women were excluded; 11 did not meet the inclusion criteria, 11 did not have good quality embryos on the day of embryo transfer, 2 for previous inclusion in the same study in a previous cycle, and 5 had poor quality video image which interfered with accurate assessment of the peristaltic waves (Fig. 1).

Women with a median age of 31 years made up most of the study cohort, whereas the mean age of their partners was 37 years. Approximately 69% had never undergone a cesarean section. The prevalence of infertility was split evenly between primary (52%) and secondary (48%) kinds, with the most frequent reasons being anovulation (19.6%) and male factor (44.8%). Most participants were going through their first round of ART. With a median AMH of 2.1 ng/mL, the hormonal profiles (FSH, LH, AMH, TSH, and prolactin) were within the predicted ranges for a population receiving ART, suggesting a mostly intact ovarian reserve (Table 1).

**Table 1:** Baseline characteristics

Variable	Result (*N* = 127)
Age (years) – median (IQR)	31 (26–35)
Husband age (years) – median (IQR)	37 (32–42)
BMI (kg/m^2^) – median (IQR)	28.5 (24.4–32.7)
Parity – no. (%)	
- Nulliparous	87 (68.5%)
- Primiparous	36 (28.3%)
- Multiparous	4 (3.1%)
Previous cesarean delivery – no. (%)	
- No	88 (69.2%)
- One or more	39 (30.8%)
Duration of infertility (years) – median (IQR)	5 (3–8)
Type of infertility – no. (%)	
- Primary	66 (52%)
- Secondary	61 (48%)
Cause of infertility – no. (%)	
- Male factor	57 (44.8%)
- Ovarian factor: (Anovulation)	25 (19.6%)
: (Low ovarian reserve)	2 (1.5%)
- Tubo-peritoneal	12 (9.4%)
- Mixed factors	10 (7.8%)
- Unexplained	21 (16.5%)
Previous ART cycles – no. (%)	
- First cycle	56 (44%)
- Previous one cycle	40 (31.5%)
- Previous two or more cycles	31 (24.5%)
Basal serum FSH (mIU/mL) – median (IQR)	5.8 (4.6–6.9)
Basal serum LH (mIU/mL) – median (IQR)	3.8 (3.0–5.5)
Serum AMH (ng/mL) – median (IQR)	2.1 (1.4–4.4)
Serum TSH (ng/mL) – median (IQR)	1.8 (1.3–2.4)
Serum PRL (ng/mL) – mean ± SD	17.15 ± 8.37

*AMH* Anti-mullerian hormone, *ART* Assisted reproductive technology, *BMI* Body mass index, *FSH* Follicle stimulating hormone, *IQR* Inter-quartile range, *LH* Luteinizing hormone, *PRL* Prolactin, *TSH* Thyroid stimulating hormone

The majority of the 127 cycles (59.8%) involved fresh embryo transfers, and the most common procedure for fresh cycles was an antagonist protocol (75%). Hormone replacement therapy (HRT) accounted for 98% of frozen transfers. A sufficient ovarian response was demonstrated by the median of 11 oocytes recovered, including 8 mature oocytes. On day three or four, the majority of the women (60.7%) had two embryos transplanted. The median endometrial thickness was 8 mm, and most embryo transfers (93.7%) were technically simple (Table 2).

**Table 2:** IVF cycle characteristics

Variable	Result (*N* = 127)
Cycle type – no. (%)	
- Fresh embryo transfer	76 (59.8%)
- Frozen embryo transfer	51 (40.2%)
Down-regulation protocol – no. (%)	
*Fresh cycles (*n* = 76)	
- Antagonist	57 (75%)
- Long agonist	5 (6.6%)
- Short agonist	13 (17.1%)
- Progestin-primed	0 (0%)
- None (in hypogonadotropic hypogonadism patient)	1 (1.3%)
*Frozen cycles (*n* = 51)	
- Programmed cycles	50 (98%)
- Ovulation induction cycles	1 (2%)
Total gonadotropin dose (IU) – median (IQR)	3000 (2500–3600)
No. of oocytes retrieved – median (IQR)	11 (7–21)
No. of mature oocytes – median (IQR)	8 (5–15)
Transferred embryos– no. (%)	
• 1 embryo	18 (14.2%)
• 2 embryos	77 (60.7%)
• 3 embryos	32 (25.1%)
Day of embryo transfer – no. (%)	
- Day 3	63 (49.6%)
- Day 4	52 (40.9%)
- Day 5	12 (9.5%)
Endometrial thickness (mm) – median (IQR)	8 (8–11)
Difficult embryo transfer – no. (%)	
- Easy	119 (93.7%)
- Difficult	8 (6.3%)
Biochemical pregnancy – no. (%)	48 (37.7%)
Clinical pregnancy – no. (%)	42 (33.1%)
Miscarriage – no. (%)	15 (11.8%)
Live birth – no. (%)	27 (21.2%)
Ectopic pregnancy – no. (%)	0 (0%)

*IQR* Inter-quartile range, *IU* International unit

Biochemical pregnancy rate was 37.7% and clinical pregnancy rate was 33.1%. The miscarriage rate was 11.8%, while 21.2% of patients had a live birth. There were no reports of ectopic pregnancies (Table 2).

The median number of uterine peristalsis waves was three in three minutes (1 per minute). Those with moderate (> 2) or low (≤ 2) peristalsis frequency had somewhat greater clinical pregnancy rates than those without peristalsis. But according to *p* = 0.43, this difference was not statistically significant. This shows that although uterine peristalsis may differ from patient to patient, in this cohort, its frequency was not significantly linked to clinical pregnancy outcomes (Table 3).

**Table 3:** Uterine peristalsis

Variable	Result (*N* = 127)
Frequency in 3 min – median (IQR)	3 (0–6)
Peristalsis per minute – media (IQR)	1 (0–2)
Peristalsis Direction – no. (%)	
- Absent	33 (26.0%)
- Cervico-fundal	62 (48.8%)
- Fundo-cervical	6 (4.7%)
- Random	26 (20.5)

The relationship between uterine peristalsis quantity and direction and clinical IVF results, clinical pregnancy, miscarriage, live birth, and biochemical pregnancy is displayed in Table 4. None of the research results were affected in a way that was statistically significant. Similarly, there was no discernible impact of either peristalsis number or direction on the research outcomes when women were subgrouped into fresh and frozen cycles (Supplementary Table 1). Despite the absence of statistical significance, pregnancy rate in women having fundo-cervical direction was zero.

**Table 4:** Relation between peristalsis and clinical IVF outcomes

Variable	*N*	Positive outcome No (%)	Chi square	*P* value
Clinical pregnancy				
Peristalsis			0.97	0.616
- Absent	34	9 (26.3%)		
- < 3	72	26 (36.1%)		
- ≥ 3	21	7 (33.3%)		
Peristalsis Direction			4.30	0.23
- Absent	34	9 (26.3%)		
- Cervico-fundal	62	22 (35.4%)		
- Fundo-cervical	5	0 (0.0%)		
- Random	26	11 (42.3%		
Miscarriage				
Peristalsis			0.70	0.71
- Absent	34	5 (14.7%)		
- < 3	72	7 (9.7%)		
- ≥ 3	21	3 (14.3%)		
Peristalsis Direction			5.82	0.12
- Absent	34	5 (14.7%)		
- Cervico-fundal	62	4 (6.5%)		
- Fundo-cervical	5	0 (0.0%)		
- Random	26	6 (23.1%)		
Live birth				
Peristalsis			3.02	0.221
- Absent	34	4 (11.8%)		
- < 3	72	19 (26.4%)		
- ≥ 3	21	4 (19,0%)		
Peristalsis Direction			5.48	0.140
- Absent	34	4 (11.8%)		
- Cervico-fundal	62	18 (29.0%)		
- Fundo-cervical	5	0 (0.0%)		
- Random	26	5 (19.2%)		
Biochemical pregnancy				
Peristalsis			0.46	0.80
- Absent	34	12 (35.3%)		
- < 3	72	29 (40.3%)		
- ≥ 3	21	7 (33.3%)		
Peristalsis Direction			3.52	0.32
- Absent	34	12 (35.3%)		
- Cervico-fundal	62	25 (40.3%)		
- Fundo-cervical	5	0 (0.0%)		
- Random	26	11 (42.3%)		

## Discussion

This prospective cohort study did not show any negative effect of uterine peristalsis on any of the clinical outcomes of IVF, neither in number nor in direction. The results did not differ in fresh or frozen cycles.

Infertility remains a challenge for both patients and clinicians. Unexplained infertility is considered one of the major challenges. Giving no reason to explain the delay of pregnancy is sometimes not easy to understand by the patients. There has been several factors proposed to be causes of unexplained infertility, but none of them has strong evidence to be linked directly to unexplained infertility [10]. One of these factors is abnormal uterine peristalsis [1].

There has been emerging evidence that the more uterine peristaltic waves may negatively affect ICSI outcomes. Vidal et al. conducted a meta-analysis including five studies that calculated uterine peristalsis at or before embryo transfer [2]. The analysis found significant decrease in clinical pregnancy rates in women having three or more uterine peristalsis per minute than those having lower peristalsis rate (OR 0.49, 95% CI 0.37–0.64). However, the included studies were heterogenous, and the results of this meta-analysis should be dealt with cautiously.

Zhou et al. found that pregnancy rate was lower in women having more than two peristalsis per minute and dramatic fall in pregnancy rates when the peristalsis were more than three [9]. Masroor et al. conducted a similar study in women undergoing frozen embryo transfer [3]. They reported that pregnancy rate in women with 4 or more peristaltic waves per minute was lower than those who had 2 or less waves (8% versus 60%, respectively). These results contradict our results which found no difference in pregnancy rate in women having either absent, less than or more than two peristalsis per minute.

Our results were similar to those of the study conducted by Hoi Sze Chung et al., who found no significant difference in clinical pregnancy between low and high peristalsis groups 47.6% versus 36.0%, respectively [11]. They assessed peristalsis at two other time spots (five minutes and one hour after embryo transfer), which resulted in significantly lower pregnancy rates in both time spots. This may highlight the role of the technique of embryo transfer itself. It is well known that vigorous transfer techniques or difficult introduction of the catheter may stimulate vigorous uterine peristalsis hindering implantation, and that may cause dramatic decreased pregnancy rate. Our study showed no impact of the peristalsis direction on the clinical outcomes, which was consistent with our results.

The direction of uterine peristalsis is another factor that is believed to affect embryo transport and implantation, thus affecting pregnancy rates [1]. Under the effect of estrogen, the uterine peristaltic waves are directed retrograde towards the uterine fundus to facilitate sperm transport during the late follicular phase. While in the luteal phase under the effect of progesterone, there is relative uterine quiescence that may help the embryo to implant and sustain pregnancy if occurred. Finally, during the menses, there are strong antegrade contractions towards the cervix to aid in shedding of the endometrium [12]. Based on these observations, it was hypothesized that fundo-cervical uterine peristalsis may hinder implantation and maintenance of pregnancy [13]. Nevertheless, very few studies in the literature assessed their role in implantation during IVF.

Kim and colleagues observed significantly lower pregnancy rates in women having fundo-cervical peristaltic wave direction than those with cervico-fundal direction [14].

Similarly, Chung et al. found significantly higher pregnancy rates in women having absent or intermediate peristalsis than those with fundo-cervical peristalsis [11]. This observation was found when the uterine peristalsis was assessed 60 min after transfer. However, when it was assessed 5 min before embryo transfer, which resembles the time frame in our study, there was no significant difference in pregnancy rates for any direction. These results aligned with the results of our study which found no impact of peristalsis direction on any of the clinical outcomes.

It is worth to mention that none of the women who had fundo-cervical peristalsis in our study got pregnant. However, there was no statistical significance due to the low number of women (*N* = 5). Although this observation may be attributed to chance, the potential negative impact could not be ruled out given that this direction was observed during menses which might be tremendous to embryo implantation. More studies are needed assessing the effect of peristalsis direction on IVF outcomes.

### Study strengths

This study was pragmatic that included all women undergoing IVF for any cause of infertility, using any protocol for down regulation aiming to study the effect of uterine peristalsis on all cohorts undergoing IVF. Additionally, we included both fresh and frozen cycles and analyzed each of these cohorts separately, which showed the same results.

In this study, uterine peristalsis was assessed on the same day of embryo transfer, just minutes before the procedure. This would reflect the direct effect of peristalsis on implantation and pregnancy rates.

The ultrasound done to assess peristalsis was performed in all women by the same operator using the same ultrasound machine to prevent inter-observer performance bias. The evaluation of the recorded ultrasound was done by two authors independently, and conflicting results were discussed and agreed upon by both authors.

### Study limitations

One of the study limitations was that the sample size was calculated based on the assumption that pregnancy rate would drop by 50% (from 50% to 25%). Therefore, a smaller effect of peristalsis on pregnancy rate would be missed. However, the power of the calculated sample size was set to 90%, which would decrease type II error of false negative results. Another limitation was the low pregnancy rate (33%) and live birth rate (21%) in the studied cohort. However, these results are consistent with other studies performed in the same center in different times [15, 16]. The method used to quantify uterine peristalsis in this study is subjective, which resulted in inter-observer variations in some of the assessed videos. However, other methods used to assess uterine peristalsis are either expensive such as cine magnetic resonance imaging [4], or invasive and cannot be used at the time of embryo transfer such as electrouterograph [17].

## Conclusions

This observational study showed no negative impact of either the number nor the direction of uterine peristalsis at the time of embryo transfer on clinical pregnancy or any other clinical outcomes of IVF. More studies are needed to confirm these results and to explore potential patient characteristics that may impact the outcomes.

## Supplementary Information

Supplementary Material 1.

## Data Availability

The datasets used and/or analyzed during the current study are available from the corresponding author on reasonable request.
