# Supplementary material for: Effect of uterine peristalsis at the time of embryo transfer on the outcomes of IVF cycles: a prospective cohort study
Source: Reprod Biol Endocrinol. 2026 Jul 9;24:72. doi: 10.1186/s12958-026-01587-0 (PMC13352654; doi:10.1186/s12958-026-01587-0)
Supplement: Supplementary file 1 [file 12958_2026_1587_MOESM1_ESM.docx]

Supplementary Table 1: Relation between peristalsis and clinical pregnancy in fresh and frozen cycles

| Variable | N | Positive outcome No (%) | Chi square | P value |  |
| --- | --- | --- | --- | --- | --- |
| Fresh cycles (n=76) |
| Peristalsis Absent <3 ≥3 | 24 43 9 | 4 (16.6%) 11 (25.6%) 2 (22.2%) | 0.71 | 0.70 |  |
| Peristalsis Direction Absent Cervico-fundal Fundo-cervical Random | 24 31 3 18 | 4 (16.6%) 7 (22.6%) 0 (0.0%) 6 (33.3%) | 2.56 | 0.47 |  |
| Frozen cycles (n=51) |
| Peristalsis Absent <3 ≥3 | 10 29     12 | 5 (50.0%) 15 (51.7%) 5 (41.6%) | 0.35 | 0.84 |  |
| Peristalsis Direction Absent Cervico-fundal Fundo-cervical Random | 10 31 2      8 | 5 (50.0%) 15 (48.4%) 0 (0.0%) 5 (62.5%) | 2.51 | 0.47 |  |
